# DeepMicroGen: a generative adversarial network-based method for longitudinal microbiome data imputation

**DOI:** 10.1093/bioinformatics/btad286

**Published:** 2023-04-26

**Authors:** Joung Min Choi, Ming Ji, Layne T Watson, Liqing Zhang

**Affiliations:** Department of Computer Science, Virginia Tech, Blacksburg, VA 24060, United States; College of Nursing, University of South Florida, Tampa, FL 33620, United States; Departments of Computer Science, Mathematics, and Aerospace and Ocean Engineering, Virginia Tech, Blacksburg, VA 24060, United States; Department of Computer Science, Virginia Tech, Blacksburg, VA 24060, United States

## Abstract

**Motivation:**

The human microbiome, which is linked to various diseases by growing evidence, has a profound impact on human health. Since changes in the composition of the microbiome across time are associated with disease and clinical outcomes, microbiome analysis should be performed in a longitudinal study. However, due to limited sample sizes and differing numbers of timepoints for different subjects, a significant amount of data cannot be utilized, directly affecting the quality of analysis results. Deep generative models have been proposed to address this lack of data issue. Specifically, a generative adversarial network (GAN) has been successfully utilized for data augmentation to improve prediction tasks. Recent studies have also shown improved performance of GAN-based models for missing value imputation in a multivariate time series dataset compared with traditional imputation methods.

**Results:**

This work proposes DeepMicroGen, a bidirectional recurrent neural network-based GAN model, trained on the temporal relationship between the observations, to impute the missing microbiome samples in longitudinal studies. DeepMicroGen outperforms standard baseline imputation methods, showing the lowest mean absolute error for both simulated and real datasets. Finally, the proposed model improved the predicted clinical outcome for allergies, by providing imputation for an incomplete longitudinal dataset used to train the classifier.

**Availability and implementation:**

DeepMicroGen is publicly available at https://github.com/joungmin-choi/DeepMicroGen.

## 1 Introduction

The field of microbiome research, which explores the collection of microorganisms living in a certain environment using the taxonomic abundance profiles of bacteria (Castro-Nallar et al. 2015), has become popular in bioinformatics over the past few decades due to the prospect for new medical treatments (Berg et al. 2020). Previous studies have shown that the microbiome plays a crucial role in complex diseases such as diabetes, inflammatory bowel disease, colorectal cancer, and allergy outcomes (Marchesi et al. 2011; Qin et al. 2012; Kostic et al. 2014; Fujimura and Lynch 2015), which indicates the potential of human microbes as biomarkers for disease diagnosis or as therapeutic targets for treatment (Manor et al. 2020). Moreover, researchers have shown that the microbiome can be altered over time by infections or medical interventions (Faust et al. 2015). Longitudinal studies of the microbiome have provided insights into changes in the composition of the microbiome over time and the association with disease outcomes (Stewart et al. 2017). Compared with single time point data, longitudinal data provide temporal information for complex trajectories of different microbes within a community, lending to understanding of the microbiome dynamics and new methods for diagnosis and treatment of disease. However, because of uneven and differing numbers of time points along the longitudinal timeline for different subjects, a valid comprehensive analysis is difficult to perform—a large amount of data cannot be utilized due to the missing samples for some timepoints (Ridenhour et al. 2017).

Recently, deep generative models such as generative adversarial network (GAN) have been proposed to address the missing data issue. The GAN model has been widely adopted in various fields including image synthesis and text generation (Creswell et al. 2018), and has been utilized for data augmentation to improve prediction or classification tasks by reducing overfitting with good results (Moreno-Barea et al. 2020). GAN has also used for imputation of missing values for a multivariate time series based on a recurrent neural network (RNN), showing improved performance compared with widely used imputation methods. Luo et al. (2018) employed a gated recurrent unit (GRU) in GAN to model the temporal irregularity of an incomplete time series, and also proposed an autoencoder-based generator to reconstruct a new sample, i.e. closest to the original incomplete dataset, and impute the missing values (Luo et al. 2019). A bidirectional RNN with the GAN model (Gupta and Beheshti 2020) and also a GRU with the GAN model (Zhang et al. 2021) were introduced to handle missing data values for predictive classification and regression tasks.

Several GAN-based approaches have been proposed for microbiome data simulation: MB-GAN learned latent spaces from the given microbial abundances and generated simulated abundances (Rong et al. 2021). DeepBioGen captured visual patterns of sequencing profiles, generated realistic human gut microbiome profiles, and generalized classifiers for type 2 diabetes (Oh and Zhang 2021). However, all the methods focused on data augmentation for a single time point microbiome dataset. To the best of our knowledge, data imputation for longitudinal microbiome data has not been addressed.

The present work proposes a deep generative method for longitudinal microbiome data imputation, DeepMicroGen. From the input dataset composed of multiple operational taxonomic units (OTUs), features incorporating the phylogenetic relationships between the taxonomies are extracted based on convolutional neural network (CNN) modules. These features are delivered to a bidirectional RNN-based GAN model, and the imputed values are generated by learning the temporal dependency between the observations measured at different time points. The performance of DeepMicroGen is compared with that of several standard baseline methods, and DeepMicroGen shows the lowest mean absolute error (MAE) for both simulated and real datasets. In addition, DeepMicroGen improves the prediction performance for allergy outcomes, training the classifier by providing a complete longitudinal dataset via imputation.

## 2 Materials and methods

**Figure 1.**
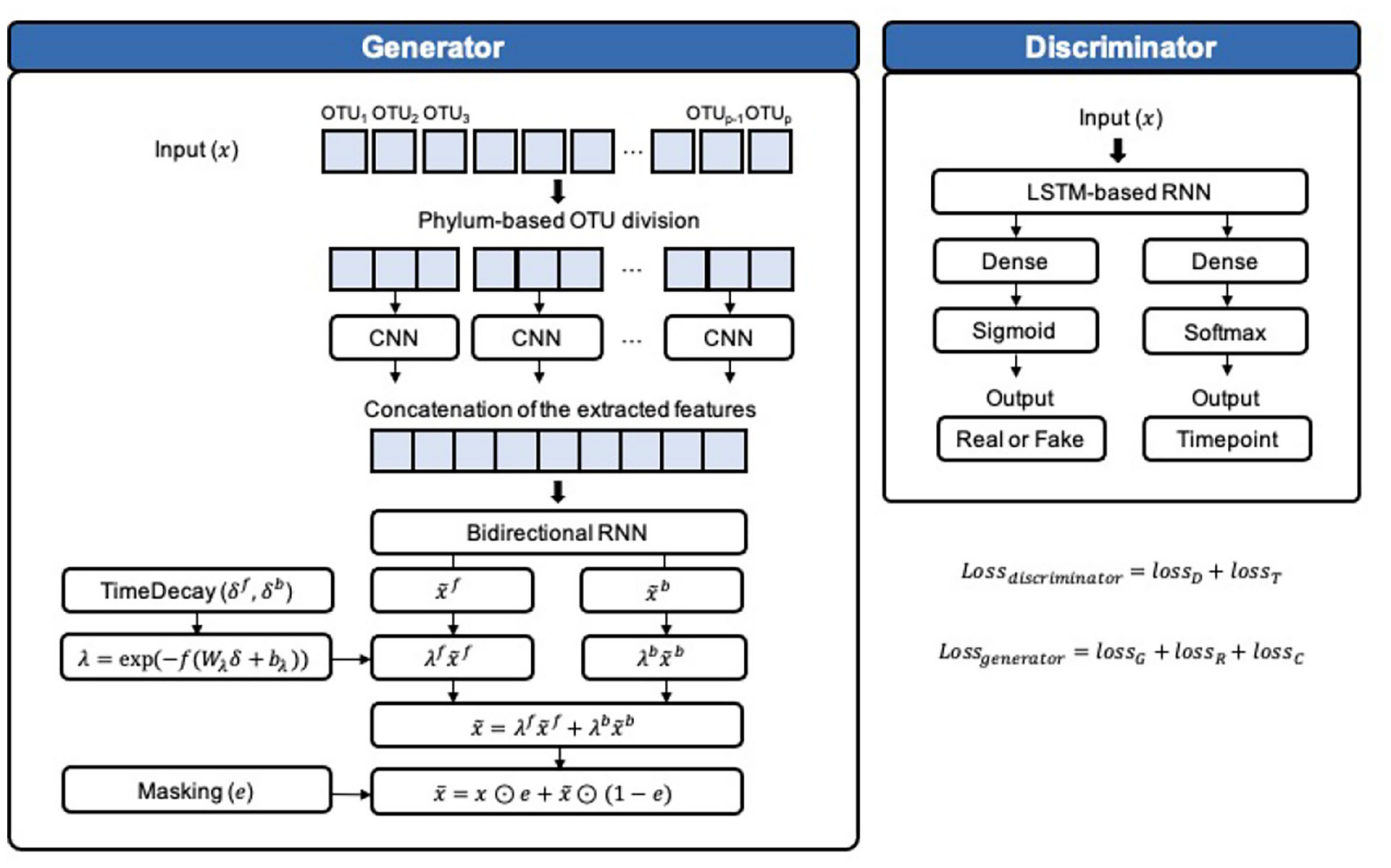
Illustration of DeepMicroGen. The generator takes actual data with missing values and imputes the missing values, while the discriminator differentiates the actual and the imputed values from the generator, and predicts the time point of each sample.

This section describes the system of DeepMicroGen, illustrated in Fig. 1.

### 2.1 Preprocessing

For longitudinal microbiome dataset imputation, species-level relative abundance (RA) profiles are given as input, which consists of real values in [0,1] indicating the percentages of the species in the total of all observed species. A pseudo-count of the minimum RA divided by two was added to zero values, and the centered log ratio (clr) transformation was performed for normalization to account for compositionality in microbiome datasets (Gloor et al. 2017).

### 2.2 Longitudinal microbiome data imputation

Let *n* be the number of taxa, $T=(t_{1}$, $t_{2}$, …, $t_{k})$ be a positive, increasing sequence of times, $x_{i}\in R^{n}$ the observations at time $t_{i}$ (where $x_{i}=0$ if the *i*th observation is missing), $x=(x_{1}$, …, $x_{k})\in R^{n\times k}$ a matrix of all the $x_{i}$, $e\in {\left\{0,1\right\}}^{k}$ a mask defined by and a time gap vector $\delta^{f}\in R^{k}$ (defined as the time lag between the current and previous values in the forward direction) given by $\delta^{b}$ is defined similarly but calculated in the backward direction, which reflects the difference between the time of the next observed value and the current time point.


$$e_{i}=\{\begin{array}{cc} 0, & \text{if}\;x_{i}\;\text{is missing},\\ 1, & \text{otherwise}, \end{array}$$

$$\delta_{i}^{f}=\{\begin{array}{cc} t_{i}-t_{i-1}, & \text{if}\;e_{i-1}=1,\;i>1,\\ \delta_{i-1}^{f}+t_{i}-t_{i-1}, & \text{if}\;e_{i-1}=0,\;i>1,\\ 0, & \text{if}\;i=1. \end{array}$$

Taking the inputs *x*, *e*, $\delta^{f}$, and $\delta^{b}$, the generator first extracts the features that capture the phylogenetic relationship using the CNN module. Authors in Sharma et al. (2020), Sharma and Xu (2021), and Wang et al. (2021) have shown that the CNN can incorporate the signals from the inherent evolutionary relationship in a local receptive field. Following Sharma et al. (2020), we divided the OTUs of input data into clusters based on the phylum. For each phylum-based cluster, Spearman correlation was measured between OTUs, constructing the Spearman rank coefficient matrix (p×p matrix for *p* OTUs in a cluster). Each row in the matrix was reduced to a geometric mean of correlation coefficients using the formula where $\rho_{{\text{OTU}}_{jp}}$ is the spearman correlation between the ${\text{OTU}}_{j}$ and the ${\text{OTU}}_{p}$.


$$\rho_{{\text{OTU}}_{j}}=\sqrt[{p}]{\left|\rho_{{\text{OTU}}_{j1}}\cdot \rho_{{\text{OTU}}_{j2}}\cdot \ldots \cdot \rho_{{\text{OTU}}_{jp}}\right|},\;1\leq j\leq p,$$

To establish the similarity between the neighboring OTUs, which would enable the CNN to capture the local features incorporating the taxonomy relationship, the OTUs were sorted in decreasing order of the geometric mean of correlation coefficients. After that, the CNN module was applied individually to each cluster and extracted the features, where the module was composed of two 1D-CNN layers with the kernel size of 3, and 16 and 8 as the numbers of filters, respectively, each followed by a Leaky ReLU activation function and a max-pooling layer. The extracted features from each cluster were concatenated and transferred to the biRNN module.

To train the temporal relations between the observations for imputing missing values, the one-layer biRNN module with tanh activation function was implemented to capture both forward and backward directions, followed by a fully connected layer, where the RNN cell is defined by where $W_{h}$ and $W_{h}^{\prime }$ are the weight matrices, $b_{h}$ is bias, and $h_{i-1}$ is the hidden state from the previous time point. From the module, two generated outputs ${\tilde{x}}^{f},{\tilde{x}}^{b}$ were obtained from each forward and backward direction, respectively. After that, each output was multiplied with the corresponding combination factors $\lambda^{f},\lambda^{b}$, and they were combined to calculate the final generated output (a n×k matrix)


$$h_{i}=\tanh(W_{h}h_{i-1}+W_{h}^{\prime }x_{i}+b_{h}),$$

$$\tilde{x}=\lambda^{f}{\tilde{x}}^{f}+\lambda^{b}{\tilde{x}}^{b}.$$

Combination factors are based on the time gap and trained as the model parameters which control the influence of the forward and backward generated values based on how far the last observed value in both directions was: where $f_{\lambda }$ is a ReLU activation function, $W_{\lambda^{f}},W_{\lambda^{b}}$ are the weight matrices, and $b_{\lambda^{f}},b_{\lambda^{b}}$ are biases. As the final imputation output $\bar{x}$, if the actual values exist in the input, the generated output $\tilde{x}$ is replaced by the actual values, and if not, the generated output becomes the final imputation output where 1 is a matrix of ones and x⊙e is a matrix whose *i*th column is $x_{\cdot i}e_{i}$.


$$\begin{array}{c} \lambda^{f}=\text{exp}(-f_{\lambda }(W_{\lambda^{f}}\delta^{f}+b_{\lambda^{f}})),\\ \lambda^{b}=\text{exp}(-f_{\lambda }(W_{\lambda^{b}}\delta^{b}+b_{\lambda^{b}})), \end{array}$$

$$\bar{x}=x⊙e+\tilde{x}⊙(1-e),$$

During the training phase, the generator was trained to minimize the loss composed of three different losses, expressed as ${\text{loss}}_{G}$ represents the classification loss where the generator is trained to maximize the probability $D(\bar{x}⊙(1-e))$ that the discriminator *D* (defined later) classifies the fake instances as actual values:


$${\text{loss}}_{\text{generator}}={\text{loss}}_{G}+{\text{loss}}_{R}+{\text{loss}}_{C}.$$

$${\text{loss}}_{G}=-\text{log}(D(\bar{x}⊙(1-e))).$$

To train the generator in a way that the outputs are close to the corresponding actual values existing in the input, define the reconstruction loss ${\text{loss}}_{R}$ as the absolute error between the actual values and the corresponding generated values:


$${\text{loss}}_{R}=\sum_{i=1}^{k}\|(x_{\cdot i}-{\tilde{x}}_{\cdot i})e_{i}{\|}_{1}/||e|{|}_{1}.$$

Moreover, the consistency loss (${\text{loss}}_{C}$) was added as the final loss term to minimize the difference between the imputed output from the forward and backward directions, defined as


$${\text{loss}}_{C}=\frac{1}{k}\sum_{i=1}^{k}\|{\tilde{x}}_{\cdot i}^{f}-{\tilde{x}}_{\cdot i}^{b}{\|}_{1}.$$

The discriminator *D* is constructed with a one layer RNN module having long short-term memory (LSTM) cells with 10 units and tahn as the activation function. The outputs of the RNN are delivered to two separate feed forward neural network models, one of which classifies the input as the real or the generated values based on the sigmoid function, the other predicts the time point for each value based on the softmax function. The discriminator is trained to optimize two losses combined as


$${\text{loss}}_{\text{discriminator}}={\text{loss}}_{D}+{\text{loss}}_{T}.$$

The probability of correctly classifying the actual values as real and the generated values as fake using a binary cross entropy loss (${\text{loss}}_{D}$) and the probability of correctly predicting the time point of each sample using cross entropy loss (${\text{loss}}_{T}$) are given by where $D(\bar{x}⊙e)$ is the probability for the actual value to be classified as real, $1-D(\bar{x}⊙(1-e))$ is the probability of the generated value being classified as fake, and y(ω) ($\hat{y}(\omega )$), ω∈{1, 2, …, k}, is the actual (model predicted, respectively) time point probability of each sample.


$$\begin{array}{c} {\text{loss}}_{D}=-\text{log}(D(\bar{x}⊙e))-\text{log}(1-D(\bar{x}⊙(1-e))),\\ {\text{loss}}_{T}=-\sum_{i=1}^{k}y(i)\;\text{log}(\hat{y}(i)), \end{array}$$

Here, DeepMicroGen was trained with the adaptive optimization algorithm Adam (Kingma and Ba 2014) with the learning rate of $10^{-3}$. For each epoch, the generator was trained after five iterations of the discriminator and the model training was stopped when the generator loss did not decrease within 1000 epochs. The loss curves during the training phase are shown in Supplementary Fig. S1. Dropout was applied to CNN layers with a rate of 0.7. The detailed architecture of DeepMicroGen is provided in Supplementary Fig. S2. DeepMicroGen was built using the Tensorflow library (Version 1.8.0).

## 3 Results

### 3.1 Experimental design

To evaluate the performance of our proposed model, we used datasets from both real studies and simulated datasets. For real study datasets, two publicly available datasets were obtained. DIABIMMUNE dataset (Vatanen et al. 2016) consists of infants recruited from three countries (Finland, Estonia, and Russia) that have substantial differences in the incidence of type 1 diabetes and allergies. From a total of 1584 stool samples based on 16s rRNA amplicon sequencing, we selected 1064 samples of 133 subjects, containing 115 OTUs at the species level for our experiments, where the samples were collected at 4, 7, 10, 13, 16, 19, 22, and 28 months. Other subjects having missing samples for those timepoints were excluded. OTU tables were generated using QIIME v1.8.0 (Caporaso et al. 2010) and Greengenes (DeSantis et al. 2006) as “the reference database.”

BONUS-CF dataset (Hayden et al. 2020) consists of the whole genome shotgun sequencing-based fecal samples of 231 infants having a sweat chloride level of at least 60 mEq/L or two well-characterized CF transmembrane conductance regulator gene mutations. Species-level RA profiles with 1374 taxa extracted by MetaPlhAn2 (Truong et al. 2015) were provided and a total of 452 samples of 113 subjects were used for our experiments, where the samples were collected at 5, 6, 8, and 10 months.

Following the longitudinal microbiome simulation method used in Sharma and Xu (2021), we simulated 200 subjects based on the DIABIMMUNE dataset. The noise was added to each OTU using a normally distributed function with mean equal to a random number in the range $\left[1\times 10^{-6},2\times 10^{-6}\right]$ and standard deviation of $10^{-6}$ to create new samples. During the simulation, it was ensured to preserve zeros and consider that the RAs add up to one, by adding or subtracting the noise term in equal proportion in each OTU set.

We selected the imputation methods in three categories for performance evaluation. First, simple imputation approaches (mean, median) were used, and time-series imputation methods composed of linear curve fitting, cubic curve fitting, and moving-window-based imputation (window-size = 3) were compared. Widely used imputation approaches for longitudinal datasets were also used: Multiple imputation by chained equations (MICE) and last observation carried forward (LOCF). In each experiment, the MAE was measured for missing samples using the clr-transformed real values and imputed values as an imputation performance. The total sum of the absolute errors between each feature for all the missing samples was obtained and the average was calculated using the “mean_absolute_error” function in Scikit-learn package (Pedregosa et al. 2011). An illustration of the experiment workflow is shown in Supplementary Fig. S3.

### 3.2 Performance evaluation of DeepMicroGen

**Figure 2.**
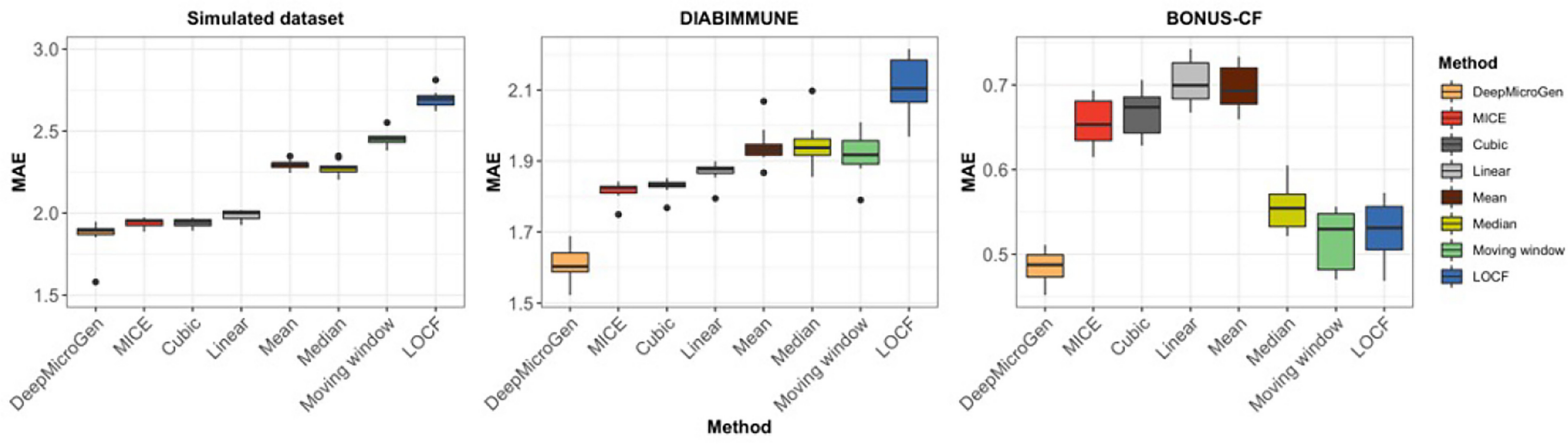
Performance comparison of DeepMicroGen with the baseline methods based on 10-fold cross-validation.

To evaluate our proposed model in several scenarios, DeepMicroGen was compared with other methods using the simulated dataset and two real datasets. Ten-fold cross-validation was performed for each dataset, where the test dataset was considered to be missing and MAE was measured. From the result (Fig. 2), DeepMicroGen outperformed the other baseline methods with the average MAE of 1.866 for the simulation dataset, where the MICE showed the second-best performance of 1.942. The other methods had an average MAE as follows: Cubic curving fitting (1.943), linear curve fitting (1.990), median (2.277), mean (2.298), moving window (2.452), and LOCF (2.698). DeepMicroGen also achieved the best imputation performance in datasets from the real studies, showing the average MAE of 1.609 and 0.486, respectively, when compared with the second-lowest average MAE of 1.816 and 0.518. These results suggest that DeepMicroGen is robust for missing sample imputation in the longitudinal microbiome dataset. As DeepMicroGen needs to optimize a large number of hyperparameters, it can be slower than other simple statistical methods. For example, the average running time of DeepMicroGen performing 10-fold cross-validation for the DIABIMMUNE dataset is 639 s, much slower than the MICE method (25 s) and the moving window method (23 s).

### 3.3 Effectiveness of each component in DeepMicroGen

DeepMicroGen utilizes GAN coupled with CNN for extracting OTU features to impute microbiome data. To see the effect of different components on imputation performance, we designed four variants of DeepMicroGen by removing or changing the components in the model. The “RNN” model eliminates both the discriminator and the CNN-based feature extraction, and the “biGAN” model removes the CNN-based feature extraction. The “AE+biGAN” model replaces the CNN with the autoencoder and the “MDeep+biGAN” model with the features encoding the phylogenetic correlation based on the method presented in MDeep (Wang et al. 2021). The 10-fold cross-validation using the DIABIMMUNE dataset was performed and the average MAE was measured for performance evaluation. Table 1 shows that compared with all four variant models, DeepMicroGen shows the best performance. Moreover, models with different feature extraction methods show highly varying imputation performance, even obtaining worse performance compared with biGAN that has no feature selection component. These results indicate the effectiveness of GAN and the feature extraction method in DeepMicroGen and that extracting features have a huge impact on longitudinal microbiome dataset imputation.

**Table 1.** Average MAE results under different neural network architectures for imputation performing 10-fold cross-validation.

Dataset	RNN	biGAN	MDeep+biGAN	AE+biGAN	DeepMicroGen
DIABIMMUNE	1.672	1.662	1.884	2.759	**1.609**
BONUS-CF	0.535	0.521	0.559	0.732	**0.474**

The best performance value for each experiment was bolded.

### 3.4 The effect of different data missing rates and mechanisms

**Figure 3.**
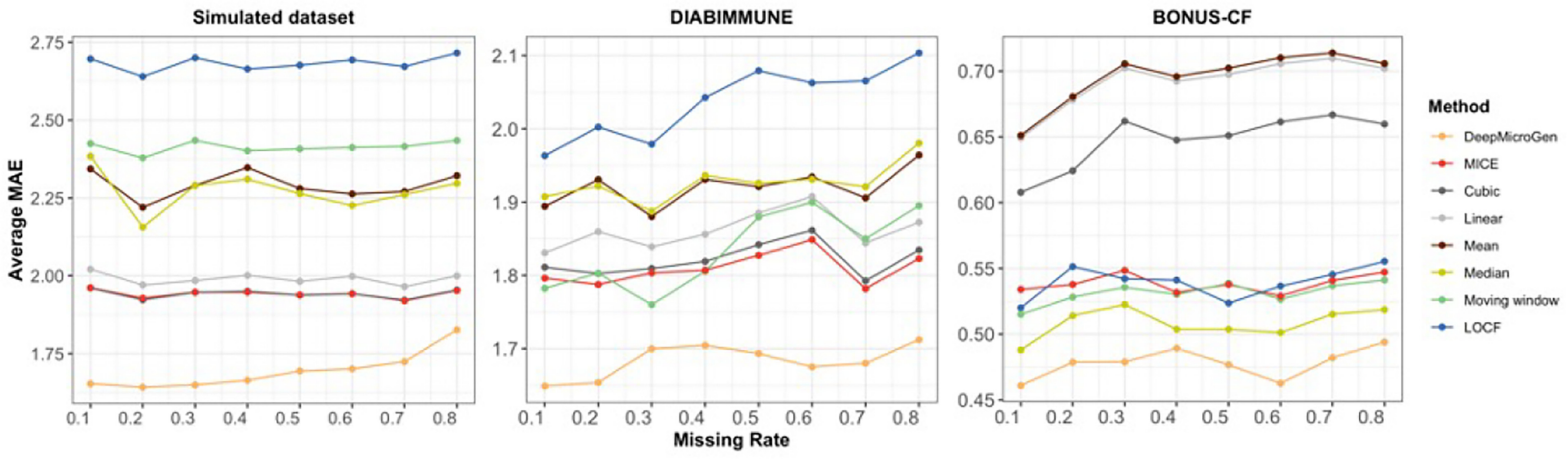
Imputation performance results with different missing rates for DeepMicroGen and other baseline methods.

When conducting longitudinal microbiome studies, due to unexpected circumstances, samples at different time points can fail to be collected and the overall data missing rate can be very high in the end. To examine how the imputation performance changes with different missing rates, we randomly discarded 10%–80% of the samples considering them as missing, and performed data imputation using DeepMicroGen. The experiment was repeated five times and the average MAE was calculated. To investigate whether DeepMicroGen could outperform the other methods for all cases, the performance of the baseline methods was also measured. The result (Fig. 3) showed that DeepMicroGen has the lowest average MAE for all the cases with different missing rates.

Missing data can be due to different mechanisms (Graham et al. 2013). The current experiment is considered to be missing completely at random (MCAR), which is often unrealistic for the longitudinal data at hand (Ibrahim and Molenberghs 2009; Liu 2015). To mimic the real world scenarios, we incorporated two missing mechanisms, missing at random (MAR) and missing not at random (MNAR) using “ampute” function in the MICE package (van Buuren and Groothuis-Oudshoorn 2011) where we can specify the missingness patterns, and performed the same experiments. This function splits dataset into multiple subsets, and each data row is a candidate for a certain missingness pattern, but whether the case will have missing values eventually depends on the missing mechanism. For MCAR mechanism, the candidates have an equal probability of becoming incomplete, and for MAR or MNAR, weighted sum scores are calculated, where these scores are a linear combination of the variables. In the case of MAR, variables that will be amputed will be weighted with 0. For MNAR, variables that will be observed will be weighted with 0. Based on its score, continuous distribution of probabilities is used to calculate whether a candidate will have missing values. Consequently, in every subset the specified proportion of data rows is made incomplete according to the missing data pattern of their candidacy. The results (Tables 2 and 3; Supplementary Table S1) showed that DeepMicroGen still had the lowest average MAE for most cases except in MNAR with the missing rate of 40%. These results support the robustness of DeepMicroGen for different experimental conditions and cases.

**Table 2.** Average MAE results with different missing data mechanisms for DIABIMMUNE dataset.

	MAR					MNAR				
Missing rate	30%	40%	50%	60%	70%	30%	40%	50%	60%	70%
DeepMicroGen	**1.600**	**1.663**	**1.755**	**1.784**	**1.741**	**1.654**	**1.623**	**1.676**	**1.752**	**1.765**
MICE	1.832	1.857	1.820	1.812	1.810	1.823	1.821	1.810	1.809	1.829
Cubic	1.841	1.868	1.835	1.825	1.818	1.837	1.829	1.818	1.823	1.842
Linear	1.876	1.900	1.870	1.869	1.866	1.870	1.863	1.869	1.873	1.865
Mean	1.852	1.937	1.891	1.976	1.902	1.925	1.915	1.907	1.941	1.912
Median	1.834	1.964	1.873	1.997	1.902	1.924	1.932	1.908	1.944	1.925
Moving window	1.788	1.861	1.834	1.908	1.798	1.855	1.857	1.869	1.893	1.818
LOCF	1.969	2.058	2.089	2.148	1.993	2.077	2.087	2.072	2.077	2.012

The best performance value for each experiment was bolded.

**Table 3.** Imputation performance results based on the average MAE for different missing data mechanisms using BONUS-CF dataset.

	MAR					MNAR				
Missing rate	30%	40%	50%	60%	70%	30%	40%	50%	60%	70%
DeepMicroGen	**0.488**	**0.491**	**0.478**	**0.478**	**0.493**	**0.461**	0.463	**0.491**	**0.502**	**0.509**
MICE	0.524	0.538	0.555	0.536	0.535	0.523	0.525	0.542	0.557	0.543
Cubic	0.642	0.651	0.659	0.643	0.651	0.623	0.629	0.656	0.668	0.652
Linear	0.687	0.693	0.701	0.688	0.695	0.666	0.674	0.700	0.710	0.696
Mean	0.690	0.696	0.704	0.693	0.700	0.669	0.676	0.704	0.713	0.699
Median	0.499	0.508	0.526	0.503	0.502	0.499	0.490	0.512	0.530	0.527
Moving window	0.491	0.495	0.531	0.508	0.510	0.480	0.471	0.513	0.516	0.525
LOCF	0.512	0.503	0.532	0.525	0.514	0.480	**0.461**	0.516	0.519	0.523

The best performance value for each experiment was bolded.

In addition, to examine whether capturing the influence of both forward and backward directions lead to better longitudinal data imputation, additional experiment was performed. We implemented a variant of DeepMicroGen which was based on unidirectional RNN considering only time gap of the forward direction, and excluding the consistency loss in the final generator loss. The results (Supplementary Table S2) showed that the bidirectional RNN models achieved better imputation performance than the unidirectional RNN models, indicating that considering the influence of both forward and backward directions lead to the improvement of the longitudinal dataset imputation.

### 3.5 Preserving the similar characteristics of missing samples in data imputation

**Figure 4.**
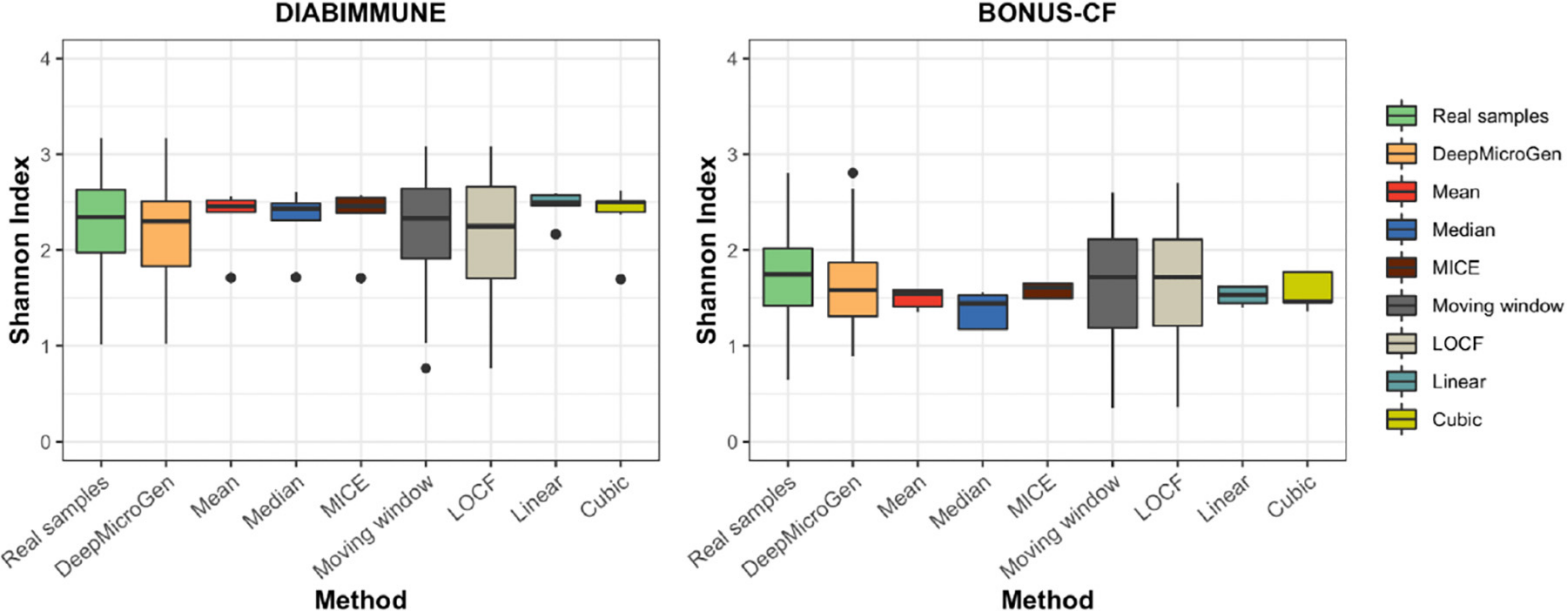
Comparison of alpha-diversity based on Shannon index measured from real samples and imputation output from DeepMicroGen and other baseline methods.

**Figure 5.**
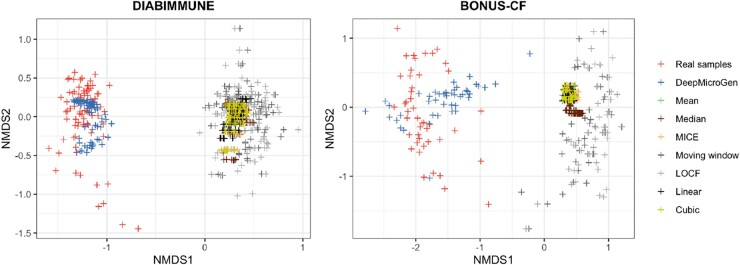
Beta-diversity visualization using NMDS for the real samples and imputation output from DeepMicroGen and other baseline methods.

DeepMicroGen takes the species-level RA profiles as input, performs the centered log-ratio (clr) transformation to the abundance values, runs DeepMicroGen models, and generates the clr-transformed imputation values for the missing samples. However, users might be also interested in RA values for downstream analyses such as alpha- and beta-diversity computation. To address this, DeepMicroGen scales the clr-transformed imputation values to RA values and set those scaled values that are smaller than the pseudo-count to zeros. To make sure that the RAs of all the OTUs sum to 1, the small difference due to this zero-setting operation is partitioned by the number of nonzero OTUs and added to each of the nonzero OTUs. One important question is whether the imputed RA profiles preserve similar characteristics to the original samples. We randomly selected 10% of the data considering them as missing and performed data imputation using DeepMicroGen and other baseline methods. Post-processing was performed on all the imputation output. For imputation output from each method, we calculated the alpha-diversity using Shannon index and compared the alpha-diversity of the real samples and the imputation output by paired *t*-test (Fig. 4). Beta-diversity was also measured based on Bray–Curtis distance and the results were compared based on the visualization using non-metric multidimensional scaling (NMDS) (Fig. 5). For comparison, we also calculated Pearson correlation coefficients between the alpha-/beta-diversities of real samples and the imputation output from each method (Tables 4 and 5). From the results, we could see that imputation output from the mean (*P*-value < .001), median (*P*-value < .001), MICE (*P*-value < .05), linear (*P*-value < .005), and cubic curve fitting (*P*-value < .005) showed significantly different alpha-diversities compared with the real dataset. The results of imputed dataset from the moving window, LOCF, and DeepMicroGen showed similar alpha-diversities to those of the real samples. However, the alpha-diversity based on the outputs of moving window and LOCF showed low correlation with the real samples, whereas DeepMicroGen achieved the highest correlation in both DIABIMMUNE and BONUS-CF dataset. For beta-diversity comparison, we could also see the overlap between the real samples and imputation output from DeepMicroGen, whereas the other methods showed little overlap with the real data.

**Table 4.** Pearson correlation coefficients between the alpha-diversity of real samples and the imputation output from each method.

Dataset	DeepMicroGen	Mean	Median	MICE	Linear	Cubic	LOCF	MW
DIABIMMUNE	**0.641**	0.344	0.410	0.329	0.399	0.326	0.133	0.217
BONUS-CF	**0.698**	–0.271	0.263	–0.277	–0.273	–0.287	0.136	0.204

The best performance value for each experiment was bolded.

**Table 5.** Pearson correlation coefficients between the beta-diversity of the samples from real data and the imputed samples from each method.

Dataset	DeepMicroGen	Mean	Median	MICE	Linear	Cubic	LOCF	MW
DIABIMMUNE	**0.364**	0.336	0.334	0.338	0.321	0.320	0.262	0.309
BONUS-CF	0.210	0.067	–0.009	0.026	**0.263**	0.147	0.099	–0.048

The best performance value for each experiment was bolded.

Furthermore, to test whether the proposed method can identify the true zero RA values during imputation, we compared the size of the symmetric difference of the sets {true zero RAs} in the real samples and {predicted zero RAs} in the imputation output (Table 6). The percentage of the true zero RAs that each imputation method correctly predicted, and the average MAE results for the non-zero RAs were also compared (Table 7; Supplementary Table S3). The MAE was calculated based on the post-processed dataset where the sum of RAs equals to 1. The number of OTUs at the species level in DIABIMMUNE and BONUS-CF datasets are 115 and 1374, respectively, where the abundance values of most of the OTUs are close to zero, the MAE also results in value lower than 0.01. In addition, the MAE based on the different taxon rank was reported to show the detailed comparison of each imputation method (Supplementary Table S4). All these results support that DeepMicroGen can capture similar characteristics of the missing samples during the imputation and can be used to impute the raw RA data effectively as well, which might be useful for downstream analysis.

**Table 6.** Comparison of the size of the symmetric difference of the sets {true zero RAs} in the real samples and {predicted zero RAs} in the imputation output from each method.

Dataset	DeepMicroGen	Mean	Median	MICE	Linear	Cubic	LOCF	MW
DIABIMMUNE	**42**	50	50	50	50	50	49	48
BONUS-CF	**263**	270	266	269	374	326	374	411

The best performance value for each experiment was bolded.

**Table 7.** The percentage of the true zero RAs that each imputation method correctly predicted.

Dataset	DeepMicroGen	Mean	Median	MICE	Linear	Cubic	LOCF	MW
DIABIMMUNE	**0.328**	0.138	0.310	0.140	0.142	0.134	0.254	0.280
BONUS-CF	0.527	0.550	0.531	0.553	0.378	0.317	0.566	**0.577**

The best performance value for each experiment was bolded.

### 3.6 Improvement of the disease prediction through data imputation using DeepMicroGen

We investigated whether utilizing the incomplete dataset through imputation based on DeepMicroGen could improve the disease prediction. In the DIABIMMUNE dataset, 141 infants had clinical information for the egg, milk, and peanut allergy outcome, where 117 infants had all samples for 8 timepoints, while the other 25 subjects had missing samples. We constructed a one-layer LSTM-based neural network classifier for each allergy, taking the clr-transformed RA profiles as input and measured the performance for the allergy outcome prediction by training the model with the addition of imputed 25 subjects by DeepMicroGen and other baseline methods. We compared the prediction performance of the same classifier without the imputed samples, measuring the area under curve (AUC) values for test datasets repeating the 5-fold cross-validation five times. For each fold, we obtained the prediction results showing the highest accuracy for test datasets, computed the AUC values using “roc_auc_score” function in “Scikit-learn” package, and reported the average AUC values for the 5-fold cross-validation. When the classifier was trained with the addition of imputed samples from DeepMicroGen, the average AUC improved from 0.566 to 0.605 for milk allergy and 0.556 to 0.638 for egg allergy (Table 8). The prediction performance for peanut allergy showed the highest increase from 0.512 to 0.612, achieving 19.5% performance improvement. The detailed results of the five times of 5-fold cross-validation for the classifiers trained with and without the imputed data using DeepMicroGen are reported in Supplementary Tables S5 and S6. Compared with the other methods, DeepMicroGen could achieve the highest improvement for all of the allergy outcome prediction. These results support that DeepMicroGen is an effective tool for filling in missing data that would otherwise be discarded from downstream prediction and the addition of the imputed data helps improve the model prediction.

**Table 8.** Average AUC results for the allergy outcome predictions of the classifier trained with the addition of the 25 imputed subjects using different methods, repeating 5-fold cross-validations five times.

Allergy	w/o Imp	With imputation							
		DeepMicroGen	Mean	Median	MICE	Linear	Cubic	LOCF	MW
Milk	0.566	**0.605**	0.589	0.589	0.556	0.550	0.585	0.584	0.563
Egg	0.556	**0.638**	0.542	0.550	0.553	0.590	0.552	0.535	0.514
Peanut	0.512	**0.612**	0.559	0.511	0.515	0.487	0.483	0.565	0.508

The best performance value for each experiment was bolded.

## 4 Discussion and conclusion

In this article, we presented a GAN-based longitudinal microbiome data imputation model, DeepMicroGen. The generator was designed to extract features capturing the taxonomy relationship based on the phylogenetic information through CNN and generate the imputed dataset using biRNN, while the discriminator was trained to differentiate the actual and the imputed values from the generator and predict the timepoint of each sample. DeepMicroGen was evaluated with the baseline methods using the simulated and real-studies datasets by increasing the missing rate and introducing the different missing data mechanisms. DeepMicroGen outperformed all other methods with the lowest average MAE and demonstrated robust imputation performance. Moreover, we investigated the improvement of the allergy outcome classifier utilizing the incomplete longitudinal microbiome dataset based on DeepMicroGen and it was shown that DeepMicroGen could support the prediction.

To the best of our knowledge, deep learning-based methods for longitudinal data imputation have been presented for specific research area such as imputation of MRI features for Alzheimer’s disease progression (Jung et al. 2019), electronic health records for patient management (Xu et al. 2020), and CT images for lung cancer risk estimation (Gao et al. 2021). To examine whether the neural network architecture of DeepMicroGen shows better imputation for microbiome dataset, we applied the most recent deep learning-based imputation model by Gao et al. (2021) to the longitudinal microbiome data and compared the performance of these models. Gao et al. implemented the model based on the partial bidirectional GAN for the imputation of longitudinal electronic medical record and lung CT images simultaneously. Ten-fold cross-validation was performed using the DIABIMMUNE and BONUS-CF dataset. We also performed an experiment where the missing rate was increased from 30% to 70% for each missing mechanism, repeated each experiment five times and measured the average MAE for clr-transformed output. The results (Supplementary Tables S7 and S8) show that DeepMicroGen achieved higher imputation performance compared with the model by Gao et al. (2021). DeepMicroGen consists of CNN modules to capture the local features of OTUs incorporating the taxonomy relationship, which would help the model to learn the longitudinal microbiome imputation considering the phylogenetic information. This could partially lead to the better imputation performance compared with Gao et al., which was also validated in Section 3.3, where the feature extraction method in DeepMicroGen showed a huge impact on longitudinal microbiome dataset imputation.

One limitation of DeepMicroGen is that it makes an implicit assumption that the samples are generated with the same time intervals. If subjects are sampled at irregular time intervals, DeepMicroGen may not perform well. Therefore, to train our model for accurate imputation, samples from a number of subjects for each time point need to be available for better generalization of DeepMicroGen. For the cases where subjects are sampled at different time intervals, we can still perform imputation by incorporating all the time points from the samples, but the accuracy could be low due to the small number of samples to be trained for each time point. However, this limitation occurs in all the comparison methods, as the baseline methods would also provide high errors due to the low number of samples that can be utilized for estimation.

Our DeepMicroGen is trained on and designed for microbiome dataset. However, this methodology can potentially be extended to other types of omics datasets. Briefly, e.g. this strategy can be trained on RNA-seq dataset, and analogous to clustering OTUs based on phylum, related genes can be identified and clustered based on the pathway information and the 1D-CNN module can then be applied individually to each cluster to extract the features that can then be concatenated and transferred to biRNN-based GAN. It can be also applied to DNA methylation data, by clustering CpG sites within the same promoter region to extract the local features based on CNN modules and deliver those to GAN for further imputation. As DeepMircoGen is specifically optimized for microbiome datasets, the models need to be trained and optimized for the specific types of omics data to achieve high performance.

We believe that our model will provide support to microbiome studies for utilizing the incomplete longitudinal dataset and help to improve the accurate prediction for the disease outcomes.

## Supplementary Material

btad286_Supplementary_Data

## Data Availability

The data underlying this article are available in the DIABIMMUNE and MicrobiomeDB, at https://doi.org/10.1016/j.cell.2016.04.007 and https://doi.org/10.1038/s41591-019-0714-x.
