# Supplementary material for: DeepMicroGen: a generative adversarial network-based method for longitudinal microbiome data imputation
Source: Bioinformatics. 2023 Apr 26;39(5):btad286. doi: 10.1093/bioinformatics/btad286 (PMC10196688; doi:10.1093/bioinformatics/btad286)

Supplementary Table S1.

Average MAE results based on different missing data mechanisms for simulated dataset.

	MAR					MNAR				
Missing rate	30%	40%	50%	60%	70%	30%	40%	50%	60%	70%
DeepMicroGen	1.884	1.889	1.892	1.891	1.887	1.821	1.883	1.887	1.889	1.891
MICE	1.954	1.952	1.945	1.953	1.935	1.932	1.954	1.916	1.941	1.925
Cubic	1.952	1.954	1.946	1.957	1.937	1.931	1.959	1.921	1.943	1.927
Linear	1.992	2.003	1.991	1.998	1.986	1.978	1.997	1.972	1.996	1.964
Mean	2.303	2.298	2.269	2.276	2.274	2.316	2.230	2.244	2.279	2.281
Median	2.326	2.261	2.224	2.243	2.268	2.298	2.181	2.215	2.248	2.281
Moving window	2.412	2.462	2.435	2.432	2.425	2.420	2.407	2.362	2.425	2.419
LOCF	2.689	2.698	2.688	2.690	2.714	2.661	2.636	2.610	2.690	2.656

Supplementary Table S2.

Average MAE results from bi-directional and unidirectional RNN-based DeepMicroGen with different missing data mechanism.

DIAMIMMUNE dataset

	MAR					MNAR					MCAR				
Missing rate	30%	40%	50%	60%	70%	30%	40%	50%	60%	70%	30%	40%	50%	60%	70%
DeepMicroGen (bi-directional)	1.600	1.663	1.755	1.784	1.741	1.654	1.623	1.676	1.752	1.765	1.573	1.589	1.616	1.642	1.741
DeepMicroGen (unidirectional)	1.635	1.676	1.777	1.821	1.764	1.662	1.697	1.702	1.768	1.773	1.635	1.676	1.677	1.721	1.764

BONUS-CF dataset

	MAR					MNAR					MCAR				
Missing rate	30%	40%	50%	60%	70%	30%	40%	50%	60%	70%	30%	40%	50%	60%	70%
DeepMicroGen (bi-directional)	0.488	0.491	0.478	0.478	0.493	0.461	0.463	0.491	0.502	0.509	0.443	0.475	0.476	0.470	0.505
DeepMicroGen (unidirectional)	0.490	0.492	0.496	0.486	0.527	0.488	0.484	0.493	0.519	0.539	0.461	0.474	0.485	0.485	0.518

Supplementary Table S3.
The average MAE results for the non-zero RAs for each imputation method.

Dataset	DeepMicroGen	Mean	Median	MICE	Linear	Cubic	LOCF	MW
DIABIMMUNE	**0.94e-02**	1.12e-02	1.18e-02	1.08e-02	1.08e-02	1.09e-02	1.59e-02	1.41e-02
BONUS-CF	**1.36e-02**	1.55e-02	1.56e-02	1.87e-02	1.54e-02	1.53e-02	2.16e-02	2.16e-02

Supplementary Table S4.
The average MAE results based on the different taxon rank for each imputation method.

DIAMIMMUNE dataset

Taxa-rank	DeepMicroGen	Mean	Median	MICE	Linear	Cubic	LOCF	MW
Phylum	**0.056**	0.073	0.066	0.073	0.073	0.073	0.071	0.067
Class	**0.048**	0.052	0.050	0.052	0.052	0.052	0.052	0.049
Order	**0.029**	0.032	**0.029**	0.032	0.032	0.032	0.032	0.030
Family	**0.014**	0.025	0.024	0.025	0.025	0.025	0.025	0.024
Genus	**0.011**	0.015	0.014	0.015	0.014	0.014	0.015	0.014
Species	**0.011**	0.012	0.012	0.012	0.012	0.012	0.013	0.012

BONUS-CF dataset

Taxa-rank	DeepMicroGen	Mean	Median	MICE	Linear	Cubic	LOCF	MW
Phylum	**0.086**	0.130	0.111	0.123	0.131	0.131	0.108	0.106
Class	**0.040**	0.058	0.050	0.057	0.058	0.058	0.041	0.050
Order	0.028	0.032	0.028	0.032	0.033	0.033	0.023	**0.022**
Family	**0.010**	0.015	0.013	0.014	0.015	0.015	0.012	0.013
Genus	**0.005**	0.006	**0.005**	0.006	0.006	0.006	0.006	0.006
Species	**0.001**	0.002	0.002	0.002	0.002	0.002	0.002	0.002

Supplementary Table S5.

AUC results for the allergy outcome predictions of the classifier trained without the addition of the imputed subjects, repeating 5-fold cross validations five times.

	1st experiment			2nd experiment			3rd experiment		
5-fold cv	milk	egg	peanut	milk	egg	peanut	milk	egg	peanut
1	0.454	0.614	0.413	0.584	0.543	0.435	0.613	0.529	0.413
2	0.613	0.647	0.413	0.643	0.674	0.413	0.714	0.700	0.870
3	0.558	0.525	0.655	0.562	0.554	0.679	0.635	0.578	0.679
4	0.500	0.642	0.500	0.617	0.475	0.726	0.517	0.500	0.476
5	0.441	0.500	0.477	0.500	0.546	0.455	0.471	0.572	0.409
Average	0.513	0.586	0.492	0.581	0.558	0.542	0.590	0.576	0.569

	4th experiment			5th experiment		
5-fold cv	milk	egg	peanut	milk	egg	peanut
1	0.555	0.586	0.478	0.668	0.529	0.370
2	0.643	0.547	0.413	0.714	0.500	0.457
3	0.535	0.495	0.476	0.535	0.608	0.702
4	0.672	0.475	0.476	0.517	0.617	0.500
5	0.441	0.474	0.500	0.441	0.474	0.409
Average	0.569	0.515	0.469	0.575	0.546	0.488

Supplementary Table S6.

AUC results for the allergy outcome predictions of the classifier trained with the addition of the 25 imputed subjects using DeepMicroGen, repeating 5-fold cross validations five times.

	1st experiment			2nd experiment			3rd experiment		
5-fold cv	milk	egg	peanut	milk	egg	peanut	milk	egg	peanut
1	0.597	0.493	0.413	0.567	0.521	0.435	0.639	0.629	0.435
2	0.769	0.647	0.391	0.513	0.574	0.413	0.643	0.621	0.413
3	0.569	0.691	0.452	0.658	0.608	0.655	0.585	0.775	0.679
4	0.544	0.599	0.952	0.672	0.758	0.952	0.544	0.758	0.952
5	0.632	0.447	0.977	0.632	0.599	0.477	0.632	0.724	0.409
Average	0.622	0.581	0.637	0.608	0.612	0.586	0.609	0.701	0.578

	4th experiment			5th experiment		
5-fold cv	milk	egg	peanut	milk	egg	peanut
1	0.555	0.642	0.435	0.597	0.536	0.326
2	0.571	0.768	0.913	0.685	0.447	0.391
3	0.635	0.715	0.679	0.612	0.662	0.726
4	0.589	0.833	0.702	0.644	0.783	0.726
5	0.525	0.546	0.932	0.525	0.546	0.477
Average	0.575	0.701	0.732	0.613	0.595	0.529

Supplementary Table S7.

Average MAE results for longitudinal microbiome data imputation with DeepMicroGen and Gao et al. performing 10-fold cross-validation.

Dataset	DeepMicroGen	Gao et al.
DIABIMMUNE	**1.609**	3.267
BONUS-CF	**0.474**	1.011

Supplementary Table S8.

Imputation performance results based on the average MAE for different missing data mechanism.

		MAR					MNAR				
Dataset	Missing rate	30%	40%	50%	60%	70%	30%	40%	50%	60%	70%
DIABIMMUNE	DeepMicroGen	1.600	1.663	1.755	1.784	1.741	1.654	1.623	1.676	1.752	1.765
	Gao et al.	3.320	3.230	3.277	3.215	3.313	3.335	3.241	3.165	3.319	3.249
BONUS-CF	DeepMicroGen	0.488	0.491	0.478	0.478	0.493	0.461	0.463	0.491	0.502	0.509
	Gao et al.	1.036	0.977	1.103	1.014	0.997	0.982	0.984	1.016	1.051	0.999

Supplementary Fig S1. The loss curves showing the generator and discriminator loss during the training phase in DeepMicroGen.

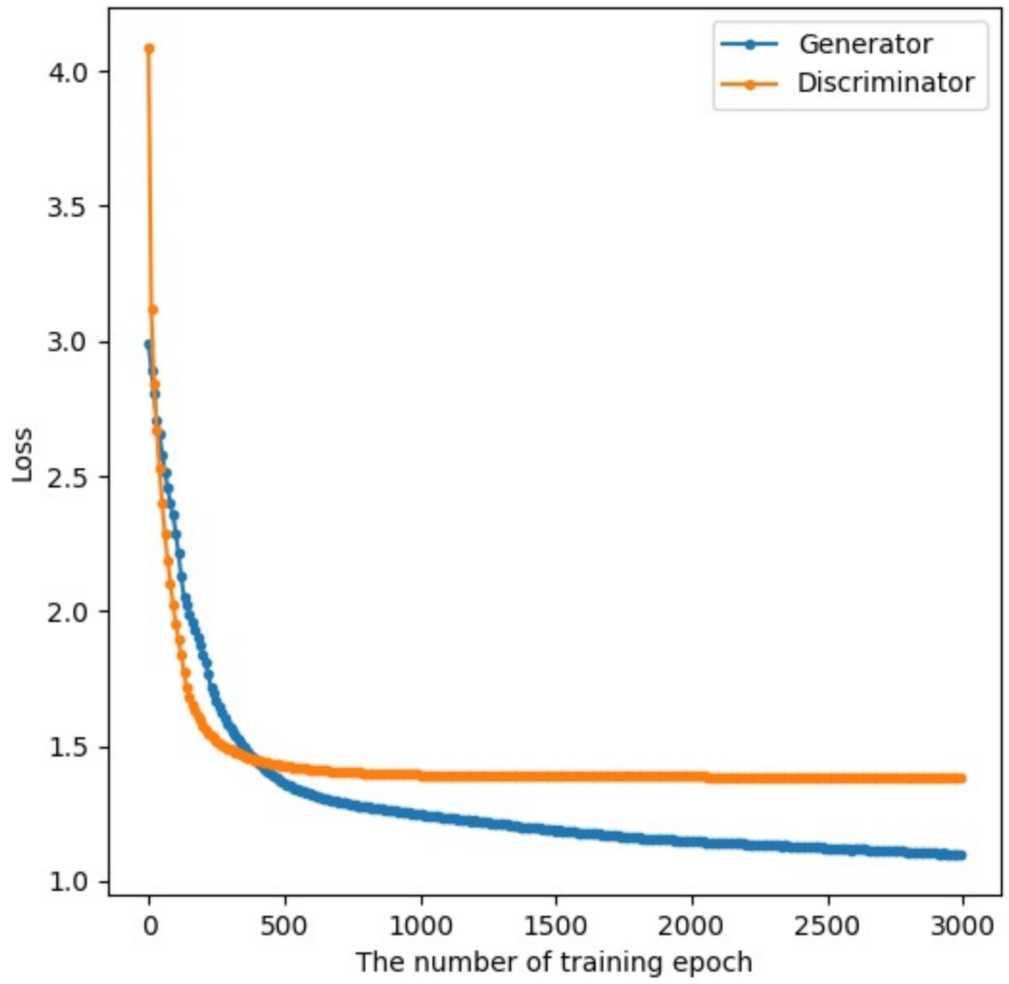

Supplementary Fig S2. Bi-directional RNN-based GAN architecture in DeepMicroGen.

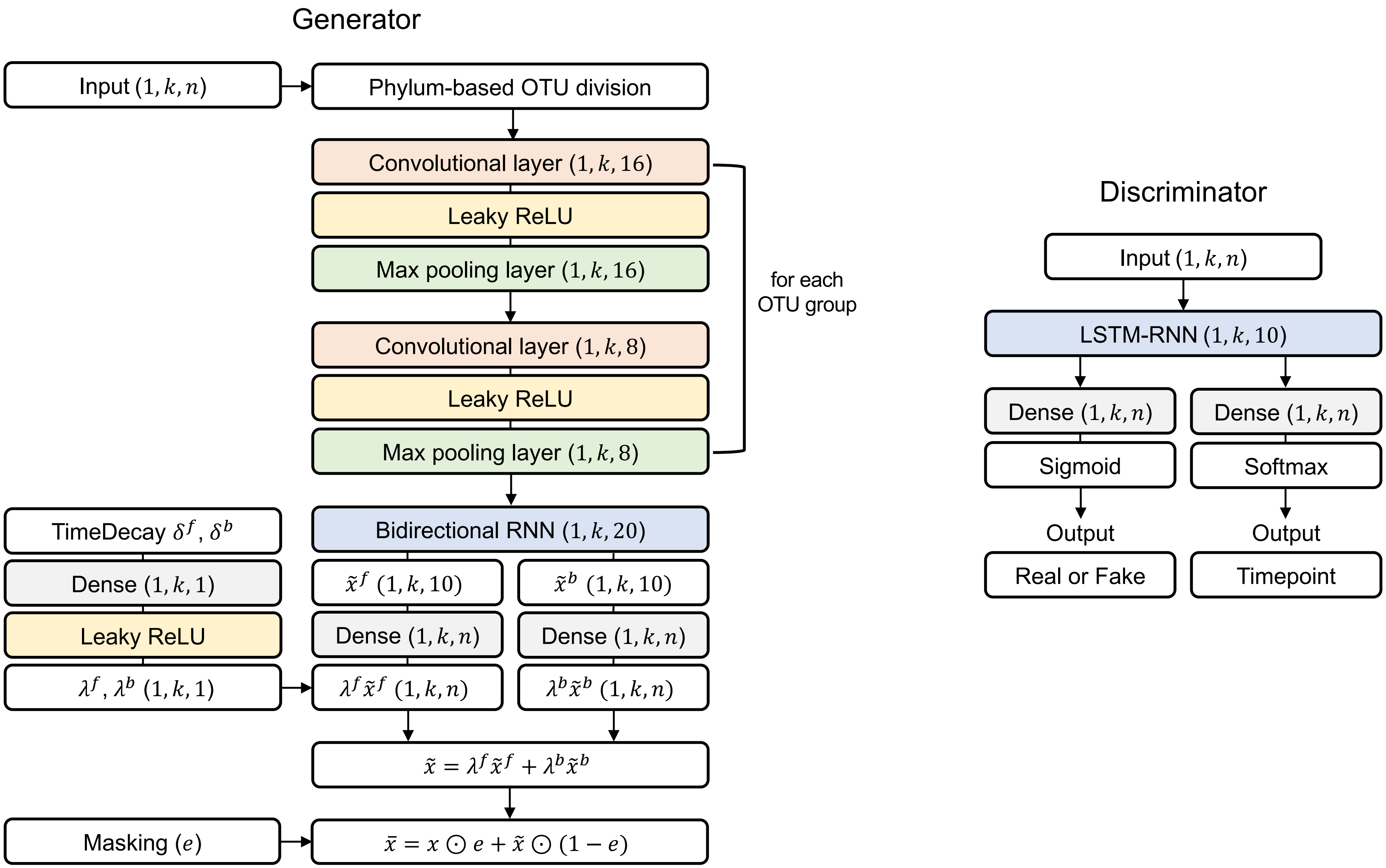

Supplementary Fig S3. Illustration of the experimental design for the performance evaluation.

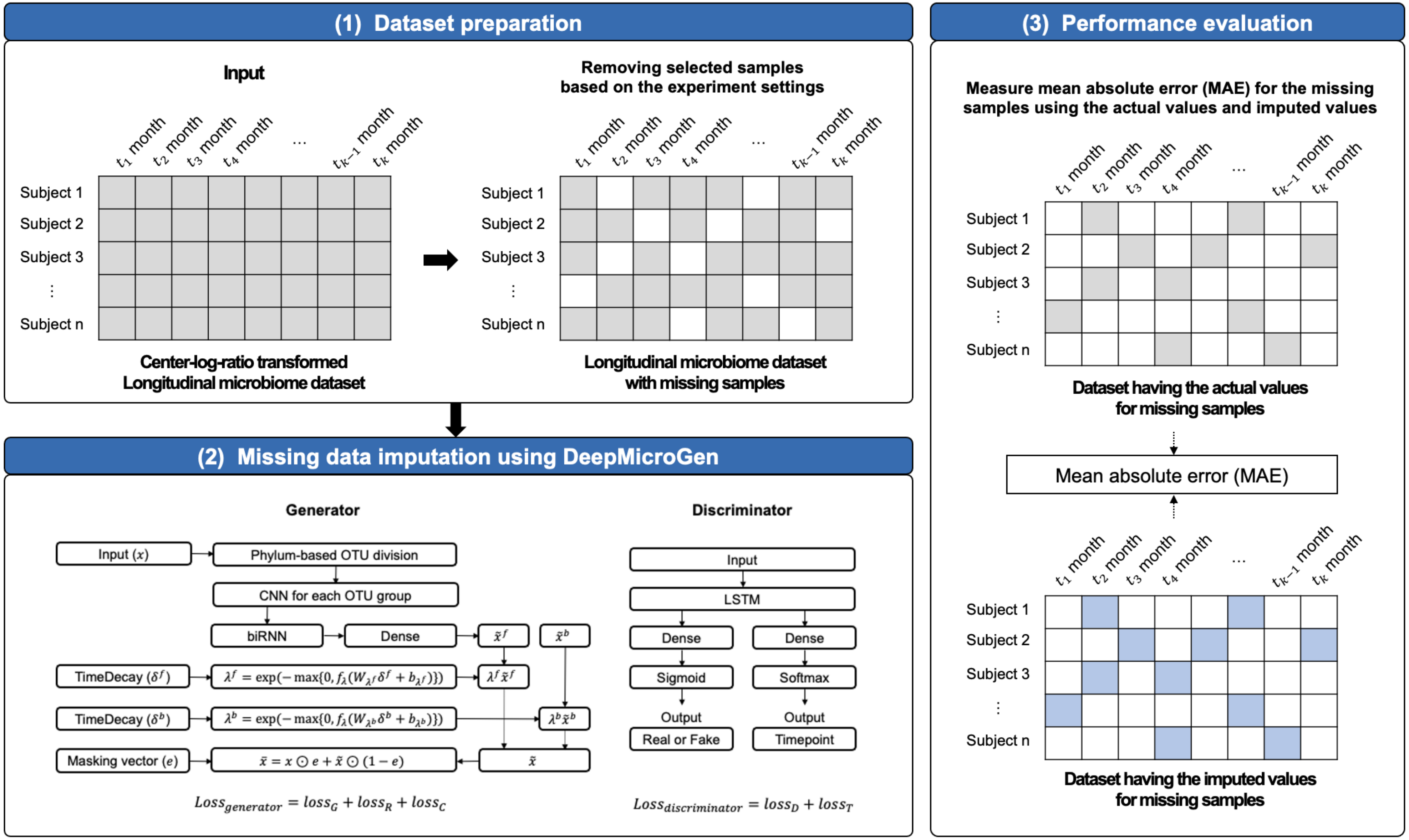

Supplement: btad286_Supplementary_Data [file btad286_supplementary_data.pdf]
